# Clones of *FeSOD*, *MDHAR*, *DHAR* Genes from White Clover and Gene Expression Analysis of ROS-Scavenging Enzymes during Abiotic Stress and Hormone Treatments

**DOI:** 10.3390/molecules201119741

**Published:** 2015-11-24

**Authors:** Yan Zhang, Zhou Li, Yan Peng, Xiaojuan Wang, Dandan Peng, Yaping Li, Xiaoshuang He, Xinquan Zhang, Xiao Ma, Linkai Huang, Yanhong Yan

**Affiliations:** College of Animal Science and Technology, Sichuan Agricultural University, Chengdu 611130, China; zhangyan1111zy@126.com (Y.Z.); lizhou1986814@163.com (Z.L.); wangxiaoj12@126.com (X.W.); Diana-Peng@hotmail.com (D.P.); 18349237779@163.com (Y.L.); 18728153879@163.com (X.H.); maroar@126.com (X.M.); huanglinkai@sicau.edu.cn (L.H.); yanyanhong3588284@126.com (Y.Y.)

**Keywords:** white clover (*Trifolium repens* L.), ROS-scavenging, expression analysis, abiotic stress

## Abstract

Increased transcriptional levels of genes encoding antioxidant enzymes play important protective roles in coping with excessive accumulation of reactive oxygen species (ROS) in plants exposed to various abiotic stresses. To fully elucidate different evolutions and functions of ROS-scavenging enzymatic genes, we isolated iron superoxide dismutase (*FeSOD*), dehydroascorbate reductase (*DHAR*) and monodehydroascorbate reductase (*MDHAR*) from white clover for the first time and subsequently tested dynamic expression profiles of these genes together with previously identified other antioxidant enzyme genes including copper zinc superoxide dismutase (*Cu/ZnSOD*), manganese superoxide dismutase (*MnSOD*), glutathione reductase (*GR*), peroxidase (*POD*), catalase (*CAT*), and ascorbate peroxidase (*APX*) in response to cold, drought, salinity, cadmium stress and exogenous abscisic acid (ABA) or spermidine (Spd) treatment. The cloned fragments of *FeSOD*, *DHAR* and *MDHAR* genes were 630, 471 and 669 bp nucleotide sequences encoding 210, 157 and 223 amino acids, respectively. Phylogenetic analysis indicated that both amino acid and nucleotide sequences of these three genes are highly conservative. In addition, the analysis of genes expression showed the transcription of *GR*, *POD*, *MDHAR*, *DHAR* and *Cu/ZnSOD* were rapidly activated with relatively high abundance during cold stress. Differently, *CAT*, *APX*, *FeSOD*, *Cu/ZnSOD* and *MnSOD* exhibited more abundant transcripts compared to others under drought stress. Under salt stress, *CAT* was induced preferentially (3–12 h) compared to *GR* which was induced later (12–72 h). Cadmium stress mainly up-regulated *Cu/ZnSOD*, *DHAR* and *MDHAR*. Interestingly, most of genes expression induced by ABA or Spd happened prior to various abiotic stresses. The particular expression patterns and different response time of these genes indicated that white clover differentially activates genes encoding antioxidant enzymes to mitigate the damage of ROS during various environmental stresses.

## 1. Introduction

In the aerobic metabolism action of plant organelles like chloroplast, mitochondria, peroxisomes *etc.*, molecular oxygen (O_2_) can be reduced to low amounts of reactive oxygen species (ROS) at various degrees. The ROS family is mainly comprised of superoxide radicals (O_2_**^·^**^−^), singlet oxygen (^1^O_2_), hydrogen peroxide (H_2_O_2_), and hydroxyl radicals (OH**^·^**^−^) [1], which act as signal molecules in the process of plant growth, development and response to environmental stress conditions. On the other hand, various stresses favor accelerated production of ROS which is rapidly capable of attacking all biomolecules including DNA, proteins and lipids. To survive complex abiotic stresses like cold, drought, salinity, heavy metal *etc.*, plants have evolved an efficient detoxification system consisting of enzymatic as well as the nonenzymatic components to scavenge excessive ROS and avoid deleterious effects [2]. In enzymatic components, superoxide dismutase (SOD) is considered to be the first and most important defense line of antioxidant enzyme systems in most organisms, which can protect plant tissues from a number of abiotic stress by catalyzing O_2_**^·^**^−^ into O_2_ and H_2_O_2_. Three SOD isoforms have been identified in eukaryotes according to their metallic cofactors, including copper zinc SOD (Cu/ZnSOD) existed in cytosol, chloroplasts and peroxisomes, iron SOD (FeSOD) located in chloroplasts and manganese SOD (MnSOD) situated in mitochondria and in peroxisomes of plant cells [3]. Differently, the functions of catalases (CAT) and peroxidase (POD) are to scavenge H_2_O_2_ accumulation in plant cells, but CAT was located in peroxisomes and POD were mainly distributed in cytosol, cell walls and vacuoles [4]. 

In addition, ascorbate peroxidase (APX), monodehydroascorbate reductase (MDHAR), dehydroascorbate reductase (DHAR) and glutathione reductase (GR) were involved in Ascorbate-Glutathione (AsA-GSH) cycle, which plays an extraordinary role in alleviating oxidative stress as well. APX is predominantly responsible for the reduction of H_2_O_2_ to H_2_O through utilizing ascorbic acid (AsA) as the deoxidizer in the chloroplast and the cytosol, simultaneously producing two molecules of monodehydroascorbate (MDHA) and a short life radical. MDHA can be converted by MDHAR or ferredoxin (Fd) of photosystem I. to AsA and dehydroascorbate (DHA). DHA is reduced to AsA by DHAR at the expense of glutathione (GSH) resulting in the production of oxidized glutathione (GSSG). The final step of this cycle is that GSSG is reduced by GR using NADPH as an electron donor. DHAR and MDHAR are physiologically important reducing enzymes in the AsA-GSH cycle for scavengaion and detoxification of ROS [5].

A great quantity of studies reported that antioxidant enzyme activities are activated in plants in response to various environmental stresses. The activated antioxidant enzymes were found in pea [6], rice [7], soybean [8], and wheat [9] under abiotic stresses such as cold, drought, salt, and heavy metals, *etc.* The resistant plant species or cultivars showed higher activities of antioxidant enzymes than the sensitive ones when exposed to the same stressful condition. For instance, As compared with the sensitive maize hybrid, the tolerant hybrid showed higher SOD, CAT, POD, APX, DHAR and GR activities in the early days of drought [10]. Furthermore, transcripts levels of *CAT*, *SOD* and *POD* were strongly induced in plants under salt, drought and flooding stress [11,12]. Under salinity stress, the transcript level of cytosolic *GR* in rice seedling was strongly up-regulated and similar results were reported in rice seedling and wheat under cold stress [13,14]. It has also been well reported that the enhanced antioxidant enzyme activities and related genes expression induced by application of phytohormones or growth regulators are closely associated with improving the tolerance of plants [12,15]. Transgenetic *Arabidopsis* lines with a c*APX*s showed higher salt tolerance than wild-type plants via the enhancement of enzymes activities including APX, CAT, SOD and GR [16]. Overexpression of *MDHAR* in transgenic tobacco increased MDHAR activity associated with enhanced tolerance to salt [17]. The concurrent overexpression of *Cu/ZnSOD*, *APX*, and *DHAR* in tobacco enhanced tolerance to various abiotic stresses [18]. Although these studies indicated that the activated gene expression together with elevated antioxidant enzymes activity could contribute to improved stress tolerance in plant, the detailed expression patterns of genes encoding antioxidant enzymes is still scarce in plants in response to different abiotic stress and plant growth regulators (PGRs)

White clover (*Trifolium repens* L.), a widely distributed legume in the world, is used for pasture hay, turfs and urban parks due to its palatability and ornamental value. However, the quality and yields of white clover are seriously affected by abiotic stresses such as drought, salinity, heavy mental stresses. Therefore, a deep insight into the mechanism of white clover in response to various environmental stresses is necessary in order to find better measures to improve tolerance to abiotic stresses. The comprehensive research on the expression patterns of primary genes related to ROS-scavenging enzymes will be helpful to further explore the biological mechanism in plants in respond to environmental stresses. In this study, three genes including *FeSOD*, *DHAR* and *MDHAR* will be isolated from white clover for the first time and the dynamically transcriptional levels of nine different antioxidant enzymes will be investigated by unsing quantitative real-time ploymerase chain reaction (qRT-PCR) when white clover are exposed to a short-term abiotic stress including cold, drought, salinity, cadmium stresses as well as exogenous abscisic acid (ABA) and spermidine (Spd) treatments. The results will contribute to better understanding of specific expression patterns of genes encoding antioxidant enzymes in response to different abiotic stresses and the regulatory role of exogenous PGRs on enzymatic antioxidant defense system based on different transcriptional levels of genes in white clover.

## 2. Results and Discussion

### 2.1. Results

#### 2.1.1. Cloning and Sequence Analysis of *FeSOD*, *DHAR* and *MDHAR* Genes in White Clover

The cloned fragments of *FeSOD*, *DHAR* and *MDHAR* Genes consist of 630, 471 and 669 bp nucleotide sequence, encoding 210, 157 and 223 amino acids, respectively. Homology analysis of three genes was listed in Table 1, and relevant information was obtained from NCBI (National Center for Biotechnology Information). The *FeSOD* sequence of white clover was the most homologous relative to its counterpart *M. sativa* and *Pisum sativum* with 94% similarity coefficient. Accordingly, both cloned *DHAR* and *MDHAR* in white clover also have the highest homology degree compared to *M*. *truncatula* and *M*. *sativa*, the approximate values are 92% and 94%, respectively. The sequence of *FeSOD*, *DHAR* and *MDHAR* have been submitted to the gene bank for verifying and the acquired accession numbers are KP202173, KP202171 and KP202172, respectively.

**Table 1:** The homology of *FeSOD*, *DHAR* and *MDHAR* comparison with other plant species.

Gene	Species	Identity(%)	Accession No.
*FeSOD*	*Medicago sativa*	94	AF377344.1
	*Pisum sativum*	94	AJ496175.1
	*Lotus japonicus*	89	AY525601.1
	*Vigna unguiculata*	85	AF077224.2
	*Glycine max*	84	NM 001250972.1
	*Fagus sylvatica*	81	DQ787261.1
*DHAR*	*Medicago truncatula*	92	DQ006811.1
	*Cicer arietinum*	91	KF276974.1
	*Lotus corniculatus*	89	DQ013362.1
	*Glycine max*	86	NM 001250000.1
*MDHAR*	*Medicago sativa*	94	JN979555.1
	*Cicer arietinum*	91	KF276975.1
	*Pisum sativum*	90	AY730589.1
	*Glycine max*	88	NM 001289382.1

#### 2.1.2. Phylogenetic Analysis

**Figure 1:**
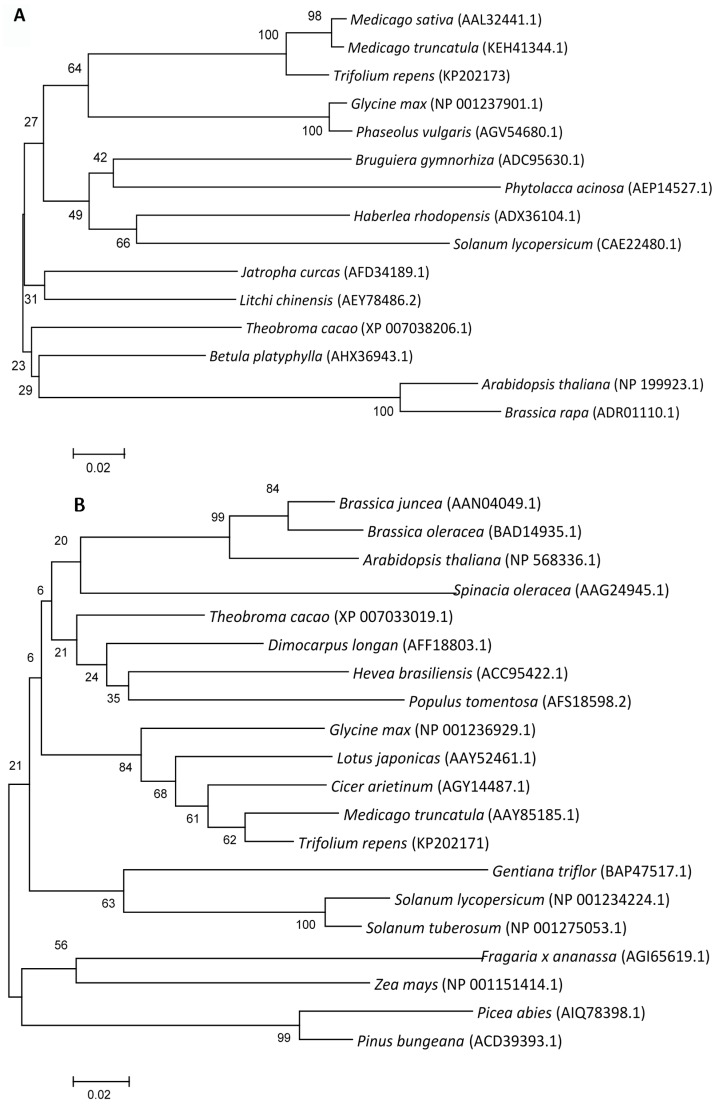

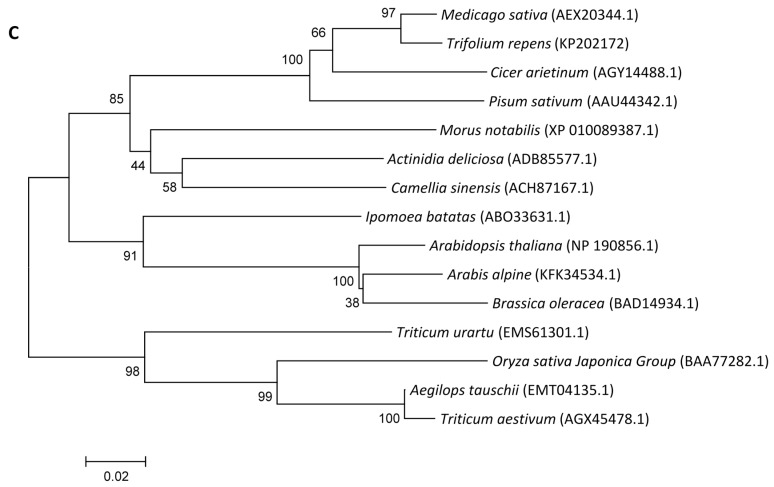
Phylogenetic tree analysis of *FeSOD* (**A**); *DHAR* (**B**) and *MDHAR* (**C**) from plants, the related information from NCBI.

Based on cloned nucleotide sequences of *FeSOD*, *DHAR* and *MDHAR*, the deduced amino acid sequence were further compared with other plant species through molecular phylogenetic tree analysis which were constructed by using MEGA software. As shown in Figure 1A, for *FeSOD*, five leguminous plants (*Trifolium repens*, two species of *Medicago* genus, *G. max* and *Phaseolus vulgaris*) were closely classified as a group in molecular phylogenetic tree of. Similar tendencies were detected in *DHAR* and *MDHAR* phylogenetic tree. For example, *T*. *repens* had the closest relation with *G*. *max*, *Lotus japonicus*, *Cicer arietinum*, *M*. *truncatula* in Figure 1B and *M*. *sativa*, *C*. *arietinum*, *P*. *sativum* in Figure 1C, respectively. Meanwhile, gramineae, cruciferous and colanaceae plants were assigned to different groups from leguminous plants. These results showed that both amino acid and nucleotide sequences of the cloned *FeSOD*, *DHAR* and *MDHAR* genes in white clover have the high degrees of homology to those derived from other plants suggesting that these three genes of antioxidant enzymes could be relatively conservative in evolution.

#### 2.1.3. Expression Patterns of Genes Encoding ROS-Scavenging Enzymes in Response to Abiotic Stress

**Figure 2:**
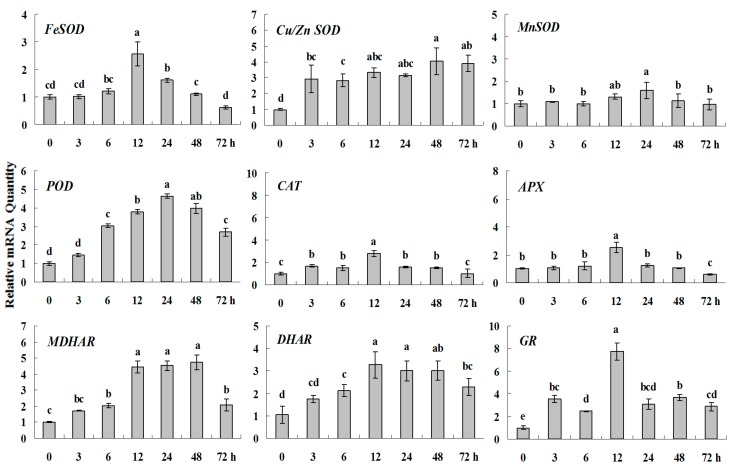
Quantitative real-time RT-PCR was used to analyze of ROS-scavenging enzyme genes expression during cold stress and normalized to β*-Actin*. White clover leaves were sampled after 0, 3, 6, 12, 24, 48 and 72 h treatment. Data represent means of three replicates. Error bars representing standard errors and the different letters above the bars represent significant difference (*p* < 0.05).

**Figure 3:**
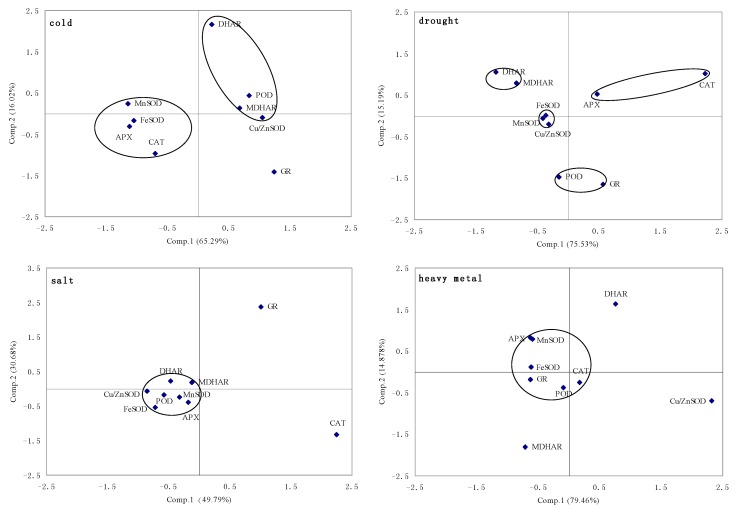

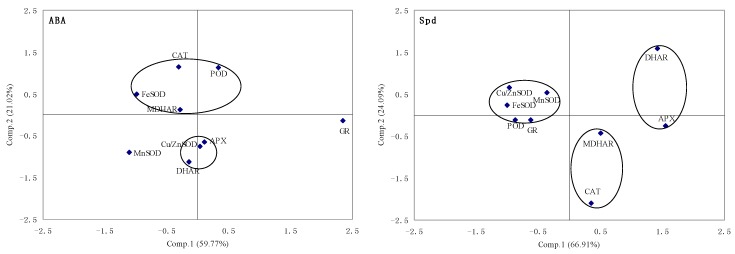
Principal components analysis of ROS-scavenging enzyme genes expression under cold, drought, salt, heavy metal, ABA and Spd.

In order to determine the expression of antioxidant genes in response to abiotic stress and plant regulators, the transcript levels of nine antioxidant enzymes were analyzed in white clover leaves exposed to cold, drought, salt, heavy metal, ABA and Spd treatments at different time points. The expression levels of *FeSOD*, *CAT*, *APX* and *MnSOD* reached their maximum at 12 h or 24 h (1.5–3 fold higher than control (0 h), but almost had no changes at other time points during cold stress. Meanwhile, the increased and persistent expressions at high levels (about 3–5 times) were observed in *Cu/Zn SOD*, *POD*, *MDHAR* and *DHAR*. Remarkably, the highest transcription level, almost 8-fold higher than control, were detected in GR (Figure 2 and Figure 3).

**Figure 4:**
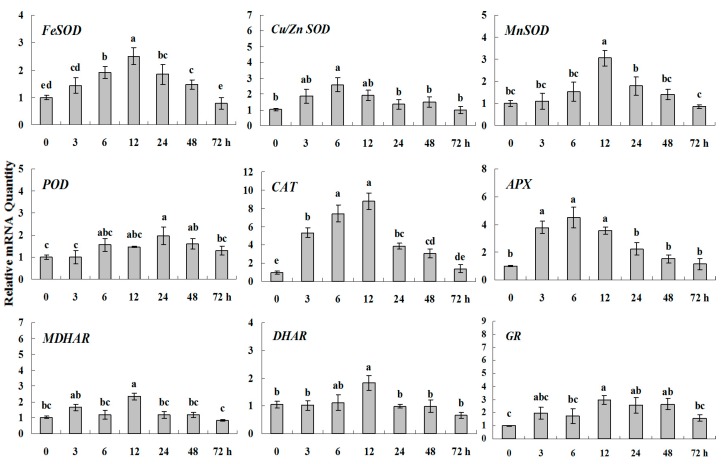
Quantitative real-time RT-PCR was used to analyze of ROS-scavenging enzyme genes expression during drought stress and normalized to β*-Actin*. White clover leaves were sampled after 0, 3, 6, 12, 24, 48 and 72 h treatment. Data represent means of three replicates. Error bars representing standard errors and the different letters above the bars represent significant difference (*p* < 0.05).

15% PEG6000 was used to simulate drought stress to analysis of the expression patterns of ROS-scavenging system genes in white clover (Figure 3 and Figure 4). *CAT*, *APX*, *FeSOD*, *Cu/ZnSOD* and *MnSOD* genes were activated quickly under drought stress at 3 h and got their highest expression at 6 h or 12 h along with a pattern of single-peak curve, among which, *CAT*, *APX* and *SOD*s showing 9-fold, 4.5-fold and 2.5–3 fold higher expression levels than the basal, respectively. However, the upgraded transcript levels of *POD*, *MDHAR*, *DHAR* and *GR* were less than 2 times during drought and their peak expression were observed at 12 h or 24 h.

**Figure 5:**
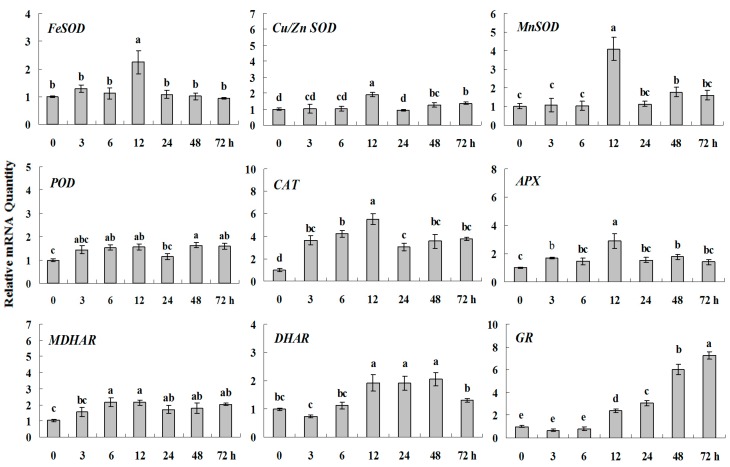
Quantitative real-time RT-PCR was used to analyze of ROS-scavenging enzyme genes expression during salt stress and normalized to β*-Actin*. White clover leaves were sampled after 0, 3, 6, 12, 24, 48 and 72 h treatment. Data represent means of three replicates. Error bars representing standard errors and the different letters above the bars represent significant difference (*p* < 0.05).

The relative expression levels of genes encoding antioxidant enzymes under salt stress were shown in Figure 3 and Figure 5. Three homologs of *SOD* genes, *POD*, *APX*, *MDHAR* and *DHAR* presented the similar transcript patterns. Their gene transcript levels reached to peak value at 12 h and showed 2–4 folds higher than control. Furthermore, salt stress induced *CAT* and *GR* genes expression obviously different from others. The highest transcript level of *CAT* was 5.5 times at 12 h than the initial level at 0 h and *GR* expression began to rise at 12 h and was maintained at higher level from 12 to 72 h, and GR was 7.5 times higher at 72 h than at 0 h.

**Figure 6:**
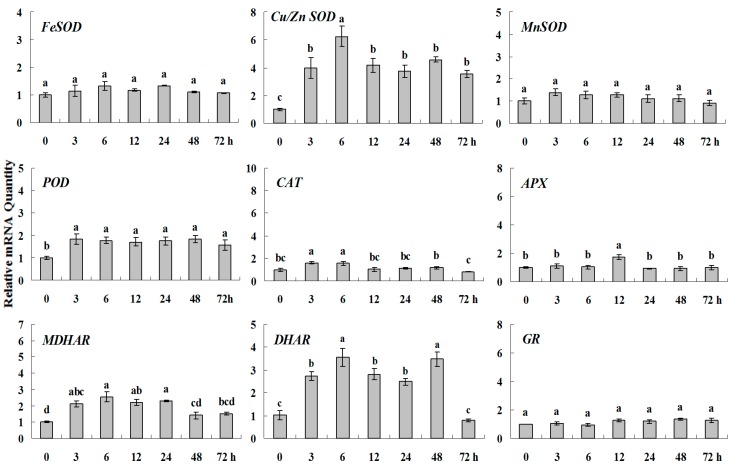
Quantitative real-time RT-PCR was used to analysis of ROS-scavenging enzyme genes expression during heavy metal stress and normalized to β*-Actin*. White clover leaves were sampled after 0, 3, 6, 12, 24, 48 and 72 h treatment. Data represent means of three replicates. Error bars representing standard errors and the different letters above the bars represent significant difference (*p* < 0.05).

The expression patterns of genes encoding antioxidant enzymes treated with 600 μM CdSO_4_ were also detected (Figure 3 and Figure 6). The gene expression levels of *FeSOD*, *MnSOD*, *POD*, *CAT*, *APX* and *GR* had only little change during heavy metal stress. As compared with *MDHAR* which showed a higher expression during 3–24 h, *DHAR* presented peaks at both 6 h and 48 h and their highest expression levels were 2.5 and 3.5 times over the initial value, respectively. In addition, *Cu/ZnSOD* was very susceptible to heavy metal. Its expression level quickly went up to and showed four times higher at 3 h than the control and reached the peak value at 6 h (6.5 times higher than control).

#### 2.1.4. Expression Patterns of Genes Encoding ROS-Scavenging Enzymes in Response to PGRs

**Figure 7:**
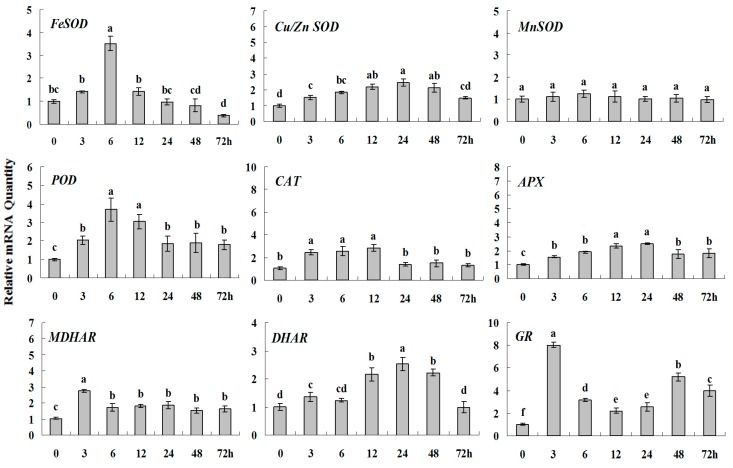
Quantitative real-time RT-PCR was used to analyze of ROS-scavenging enzyme genes expression treatment with ABA and normalized to β*-Actin*. White clover leaves were sampled after 0, 3, 6, 12, 24, 48 and 72 h treatment. Data represent means of three replicates. Error bars representing standard errors and the different letters above the bars represent significant difference (*p* < 0.05).

Figure 3 and Figure 7 showed the expression pattern of genes encoding antioxidant enzymes in response to exogenous ABA as compared to the control (0 h without ABA treatment) in different time points. *CAT*, *MDHAR*, *Cu/ZnSOD*, *APX* and *DHAR* had the similarly higher expression levels, about 2 or 2.5-fold higher than control, and the first two and latter three exhibited the peak value at 3 h and 24 h, respectively. The transcription of *FeSOD* and *POD* increasingly reached to their maximum values at 6 h, over 3.5-fold higher than control. The transcript of *GR* was up-regulated to the largest level at 3 h, nearly 8 times higher compared with no ABA treatment. *MnSOD* expression almost did not change during ABA treatment.

**Figure 8:**
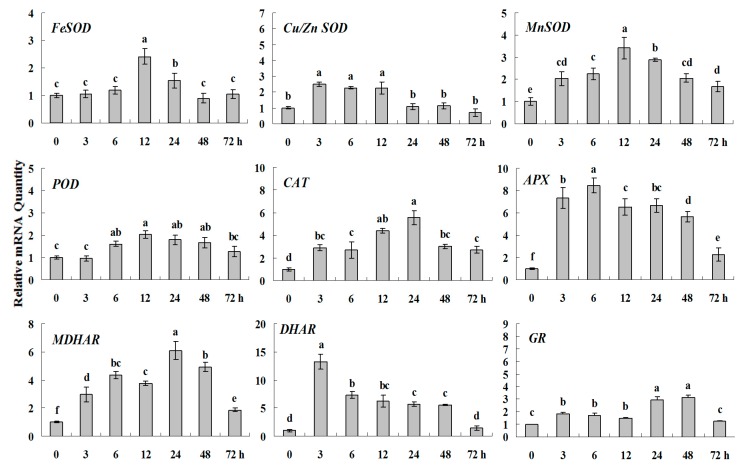
Quantitative real-time RT-PCR was used to analyze of ROS-scavenging enzyme genes expression treatment with Spd and normalized to β*-Actin*. White clover leaves were sampled after 0, 3, 6, 12, 24, 48 and 72 h treatment. Data represent means of three replicates. Error bars representing standard errors and the different letters above the bars represent significant difference (*p* < 0.05).

Under exogenous Spd, expression patterns were shown in Figure 3 and Figure 8. Except *FeSOD* and *POD*, the other genes were rapidly activated after 3 h of treatment, Firstly, the expression level of *DHAR* and *APX* was increased by Spd at 3 h or 6 h about 13- and 8.5-folds higher than control, respectively. Secondly, the supreme expression of *CAT* and *MDHAR* appeared at 24 h and increased by 5.5-fold or 6-fold, respectively. Transcript levels of *SOD*s, *POD* and *GR* enhanced by 2–3.5 fold compared to control.

#### 2.1.5. Principal Component Analysis of Genes Expression

The principal component analysis of genes expression (Figure 3) showed the transcription of *GR*, *POD*, *MDHAR*, *DHAR* and *Cu/ZnSOD* were rapidly activated with relatively high abundance during cold stress. Differently, *CAT*, *APX* and three *SOD*s exhibited more abundant transcripts compared to others under drought stress. Salt stress induced *CAT* and *GR* genes expression obviously different from others. *Cu/ZnSOD*, *MDHAR*, and *DHAR* are very susceptible to heavy metal. *Cu/ZnSOD*, *APX* and *MDHAR* have the similar expression patterns, while *FeSOD*, *CAT*, *POD* and *MDHAR* have the similar expression patterns under ABA treatment. The transcript of *GR* was up-regulated significantly compared with no ABA treatment. Three expression patterns have been divided under exogenous Spd as follows, one is three *SOD*s, *POD* and *GR*, the second type is *CAT* and *MDHAR*, and the last one is *APX* and *DHAR*.

### 2.2. Discussion

To comprehensively reveal the effects of antioxidant protection and potentially regulatory function of phytohormones in white clover in response to different abiotic stresses, *FeSOD*, *DHAR* and *MDHAR* genes were cloned in white clover for the first time through homologene sequence and their evolutionary relationships were compared with other plants. The expression profile of main genes encoding antioxidant enzymes in ROS-scavenging system were characterized by temporal variation in white clover under cold, drought, salt, heavy metal, ABA and Spd treatments. The comparative analysis of the *FeSOD*, *DHAR* and *MDHAR* genes between white clover and homologous sequences in other plants indicates that the cloned three genes are relatively conserved in the process of evolution. The deductive amino acid sequences of cloned genes also show highly conserved properties as compared to other known proteins from different species, which suggests that they may have similar functions. Researches in other plants suggest that improved cold tolerance could be induced by increased expression level of genes encoding antioxidant enzymes [14]. As well as we known, white clover derived from temperate area exhibited less cold tolerance. Under cold stress, the analysis of genes expression pattern showed all tested genes were almost up-regulated during cold stress, *GR*, *Cu*/*ZnSOD*, *POD*, *DHAR* and *MDHAR* demonstrated the abundant up-regulation in this study. especially *GR* which significantly up-regulated at most of time points through a significant analysis, but maximum can be found at 12 h implying that 12 h was *GR* maximum response time under cold stress. The improved *MnSOD*, *CAT*, *POD*, *APX*, *DHAR*, *MDHAR* and *GR* expression was also detected in wheat lines and two *Chrysanthemum* species under cold acclimation [18,19], which was similar with our results. However, the relatively steady expression of *Cu*/*ZnSOD* and *FeSOD* in above two reported plants were contrasted to white clover in our study. These data indicated that different plant species may have not only common but differential antioxidant defense mechanism in response to cold stress at molecular level.

Under drought stress, *Cu*/*ZnSOD* and *FeSOD* genes were rapidly activated at 3 h, while *MnSOD* increased significantly at 6 h and then reached to maximum at 12 h in leaves of white clover. Meanwhile, *CAT* and *APX* were more remarkably upregulated than three *SOD* genes during early stage of drought. In addition, both *POD* and *GR* remained the high transcription level during the latter period. The expression profiles of these genes were coincident with the development of their enzyme activities in white clover in response to drought stress [20,21]. Furthermore, the transcriptional activation of *MDHAR* and *DHAR* in this study further confirmed the result that their activities might be mostly affected by drought in white clover [21]. Under salt stress, the transcription of CAT was markedly activated prior to others and then kept a high level in whole period relative to GR, which showed the largest transcript in the later stage in white clover. This result was consistent with those findings that were observed in salt tolerant *M*. *truncatula* genotype [22] and in *U. fasciata* under high salt concentration [23]. Meanwhile, the transcriptional induction of other antioxidant enzymes increased differentially by salinity treatment in this work. The expression of *APX*, *POD* and *CAT* were provoked successively along with a similar trend of their enzyme activity in *Panax ginseng* seedlings [24]. The transcript abundance and enzyme activity of FeSOD, MnSOD, CAT and APX were promoted in *U. fasciata* under high salt concentration [23]. Salt stress induced transcriptional up-regulation of *Cu/ZnSOD*, *MnSOD*, *APX* and *CAT* genes and increased activity of corresponding antioxidant enzymes in rice seedlings [13]. Overexpression of *MnSOD*, *MDHAR*, *DHAR* and *CAT* in transgenic plants exhibited the improvement of salt tolerancee [25,26,27]. Complex response patterns of genes encoding antioxidant enzymes were detected in our study and other plants under salt stresses, suggesting that remarkable changes of these genes could be associated with potential salt tolerance.

Cadmium (Cd) is one of the most dangerous environmental heavy metal pollutants. Cd stress can induce the extra production of ROS resulting in the structural destruction and functional disorder of the cell [28]; therefore, antioxidative defense is an important mechanism in plants for surviving from heavy metal toxicity. The works of Manier, *et al.* [29] showed that white clover has a high plasticity in response to heavy metal stresses. In this study, the up-regulation of *MDHAR* transcription level and no obvious changes in *MnSOD*, *APX*, and *GR* were in agreement with those detected in *Pisum* *sativum* under Cd treatment. However, the most significantly activated *Cu/ZnSOD* transcription level was opposite [30]. In addition, we also found that *DHAR* and POD were transcribed obviously and moderately, respectively, and the induction of *FeSOD* and *CAT* transcription almost did not happen under Cd stress. However, the SOD, CAT, APX, POD and GR activities were significantly suppressed when white clover exposed to Cd stress [31]. The discrepancy between the gene expression of antioxidant enzymes and their activities has been also observed [30], which could be explained by the fact that Cd disturbed the uptake of other necessary metal ion like Fe, Mn, Cu and resulted in a biosynthetic reduction of enzymes containing them [32,33,34]. On the one hand, the presence of post transcriptional or translational control may bring about the lack of their connection [35].

It has been well documented that abscisic acid (ABA) and polyamine (PAs) could act as modulator in plants responding to different environmental stresses. Numerous studies have indicated low concentration of ABA or PAs treatment can effectively improve plants tolerance to stresses by inducing antioxidant defense. For instance, exogenous application of ABA elevated expression levels of *MnSOD*, *POD*, *DHAR1* and *DHAR2* in leaves of *Capsicum annuum* subjected to chilling stress [36]. Spd up-regulated transcript abundance of *FeSOD* and *MnSOD* in *U. fasciata* under hypersalinity [23]. Exogenous Spd further increased expression levels of *CAT*, *APX*, and *POD* in saline-stressed *P. ginseng* seedlings [24]. ABA activated the transcripts of *APX*2, *MDHAR*, *GR* and *DHAR*, which were involved in the ROS-scavenging through AsA-GSH cycle in *Arabidopsis thaliana* [37]. However, the report that PAs directly modulate the transcription of antioxidant enzymes is quite scarce. Cadaverine, a member of PAs, induced the expression of *Cu/ZnSOD* gene in *Mesembryanthemum* *crystallinum* [38]. Spermine (Spm) activated *POD* gene expression in tobacco plants [39]. In our study, *FeSOD*, *CAT*, *POD* and *MDHAR* were rapidly activated in quantity besides *GR*, exhibiting the highest level by ABA treatment, which upregulated the expression of *Cu/ZnSOD*, *APX* and *DHAR* at relatively low levels or late stages, leaving *MnSOD* unchanged. In addition, *DHAR* and *APX* showed the highest transcriptional levels followed by *MDHAR* and *CAT*, and three *SOD*s, *POD* and *GR* had less expression induced by exogenous Spd. Interestingly, most of genes were activated by ABA or Spd prior to the induction of abiotic stresses. These observations further confirmed that plants respond to abiotic stress associated with these two PGRs and they may improve stress tolerance in plants through stimulating antioxidant defense system at transcriptional level. 

In conclusion, the cloning of *FeSOD*, *DHAR*, *MDHAR* from white clover contributes to further study their function in plants in response to various environmental stresses. Based on the previous findings that transcriptional levels and activities of antioxidant enzymes are correlated with stress tolerance in many plant species, the obtained results in this study showed that abiotic stresses and two PGRs differently affected the transcription of antioxidant enzymes in white clover plants suggesting that these preferentially, durably and largely induced genes in ROS scavenging enzyme system may act important functions in plants in response to specific stresses. 

## 3. Experimental Section

### 3.1. Plant Materials and Growth Conditions

Seeds of white clover (cv. Ladino) were surface sterilized with mixed solutions of NaClO (0.5%, *w*/*v*) for 10 min followed by washing with sterile water four times. Afterwards, 0.1 g seeds were sown in each of plastic pots (35 cm long, 25 cm wide, 10 cm deep) filled with sterilized quartz sand. Seeds were germinated in distilled water for a week and then seedlings were cultivated in Hoagland’s solution [40] in the growth chamber with 12 h photoperiod at day/night temperature of 23/19 °C, 75% relative humidity, and 300 μmol·m^−2^·s^−1^ photosynthetic photon flux density. Seedlings at the trefoil leaf stage can be used in subsequent experiments. 

For the cold treatment, the leaves of seedlings were soaked in water at 4 °C in a light incubator. For the PEG6000, NaCl and CdSO_4_ treatments, the leaves of seedlings were dipped into 15% (*w*/*v*) PEG6000, 100 mM NaCl or 600 μM CdSO_4_ solution at 23 °C in a light incubator. For ABA and Spd treatments, leaves were dipped into solutions containing with 100 μM ABA and 20 μM Spd, respectively. The treated leaves were collected at 0, 3, 6, 12, 24, 48 and 72, respectively.

### 3.2. RNA Isolation and cDNA Synthesis

Total RNA was extracted from 100 mg leaf tissue using the RNeasy Mini Kit (Qiagen, Suzhou, China) according to the manufacturer’s instructions. The RNA was treated with DNaseI to avoid DNA contamination then the quality and quantity of the samples were detected by the NanoDrop ND-1000 Spectrophotometer (NanoDrop Technologies, Wilmington, DE, USA) at 260 and 280 nm. The first strand of cDNA was synthesized with 2 μg total RNA using the PrimerScript RT-PCR Kit (TAKARA, Dalian, China) according to the instruction manual. 

### 3.3. cDNA Sequence Isolation

The synthesized cDNA was used as a template for PCR and degenerate oligonucleotide primers were designed on the basis of known sequences of *FeSOD*, *MDHAR* and *DHAR* from other closely related plant species including *Medicago sativa* and *Glycine max*. The sequences of primers were summarized in Table 2. *FeSOD*, *MDHAR* and *DHAR* gene fragments were successfully amplified with LA-Taq (TAKARA, Dalian, China) the reaction contains 2 μL cDNA, 8 μL dNTP Mixture, 2 μL (10 μM) for each primer, 25 μL 2 × GC Buffer, 0.5 μL TaKaRa LA Taq and 10.5 μL dH_2_O. The condition were 3 min at 94 °C and 30 cycles at 94 °C for 30 s, 57 °C for 30 s and 72 °C for 1 min, followed by a final extension at 72 °C for 10 min. The amplified product was gel purified by TaKaRa PCR purification kit (TAKARA, Dalian, China). Purified PCR products were inserted into the pMD19-T vector at 16 °C for 30 min according to the following reaction system: 1 μL pMD19-T vector, 4 μL purified DNA, 5 μL enzyme solution (containing T4 DNA ligase and buffer). Then transformed the above reactions into DH5α competent cells, spread each sample on a separate LB plant containing 100 μg/mL of ampicillin and incubate all of the plates overnight at 37 °C. Subsequently, pick 10 individual isolated colonies from each plate and analyze by PCR screening with M13 primers to make sure the present of inserts (TAKARA, Dalian, China) and sequenced (Sangon, Shanghai, China).

**Table 2:** Primer sequences used in the cDNA cloning of white clover *FeSOD*, *DHAR* and *MDHAR* genes.

Gene	Forward Primer (5′-3′)	Reverse Primer (5′-3′)
*FeSOD*	TCACTGCAAAGTTTGAGCTG	ACTGCTTCCCAGGAAACAAG
*DHAR*	CTAAATGGTATAGCTTTGGTCC	GAAATCGCTGTTAAAGCTTC
*MDHAR*	TTATGCAGCAAGGGAGTTTGTG	ACTTCTTTTACCTCTCCATCGG

### 3.4. Sequence Alignment, Identity Analysis and Principal Components Analysis

For the analysis of homologs, cDNAs of cloned *FeSOD*, *MDHAR* and *DHAR* were used to search in GenBank through BLAST software available at the NCBI website [41]. The sequence alignment and identity analysis were carried out using the DNAMAN software package. For molecular phylogenetic tree analysis, the deduced amino acid sequences were constructed as compared to other plant species by using MEGA software. SPSS was used to analyze principal components among the measured variables.

### 3.5. Quantitative Real-Time RT-PCR

For real-time PCR, triplicate quantitative assays were performed with SYBR^®^ Premix Ex Taq™ (TaKaRa, Dalian, China) by using an ABI 7500 FAST real time PCR platform (Bio-Rad, Waltham, MA, USA). Each reaction contains 10 μL 2 × SYBR Premix Ex Taq, 2 μL cDNA, 7.2 μL dH_2_O, 0.4 μL for each primer in a total volume of 20 μL. The amplification of β*-Actin* was used as an internal control to normalize all data. Primers used for RT-PCR are presented in Table 3. The conditions of the PCR protocol were as follows: 5 min at 95 °C and 40 repeats of denaturation at 95 °C for 15 s, annealing at 58 °C (β-*Actin*, *Cu/ZnSOD*, *FeSOD*, *CAT*, *APX*, *DHAR*, and *GR*) or 64 °C (*MnSOD*, *POD* and *MDHR*,) for 45 s, following by heating the amplicon from 60 to 95 °C to obtain the melting curve. The relative difference in expression for all genes was calculated according to the equation 2^−ΔΔCt^ [42].

**Table 3:** Primer sequences and their corresponding GeneBank accession numbers used in real time quantitative PCR.

Gene	Accession No.	Forward Primer (5′-3′)	Reverse Primer (5′-3′)
β-*Actin*	JF968419	TTACAATGAATTGCGTGTTG	AGAGGACAGCCTGAATGG
*FeSOD*	KP202173	ACACGATTTCTCAGGGTTACGAC	GCGGCCAAGACTATCAGTTCCAT
*CuZnSOD*	JQ321597.1	AACTGTGTACCACGAGGACTTC	AGACTAACAGGTGCTAACAACG
*MnSOD*	JQ321598.1	TAAGGGAACCTACCCGATAACT	CCAGGACCAAACGTCACCAAAG
*CAT*	JQ321596.1	AACAGGACGGGAATAGCACG	ACCAGGTTCAGACACGGAGACA
*POD*	AJ011939.1	TCTAGGGCAACGGTTAATTCATTC	GGTACGGATTTTCCCATTTCTTG
*APX*	JQ321599.1	TAAAGATAGTCAACCCACCTCAACA	ACCAGTCTTGGGAAACAACGTA
*DHAR*	KP202171	TGGTTACCTCCCGACCCTAT	TCTTACCAAGGAACTTTAGTCAGG
*MDHAR*	KP202172	CCAACTGCCTAAAGCCACATCT	GAAGAAAGGAAACTAACGGAGCA
*GR*	JQ321602.1	TAAACTTCCACTCCCTTTCTATCG	CTACAATATGGGTTGAGGACAGGT

## Abbreviations

ABA, abscisic acid; *APX*, ascorbate peroxidase; AsA-GSH cycle, ascorbate-glutathione cycle; AsA, ascorbic acid; *CAT*, catalase; *Cu/ZnSOD*, copper zinc *SOD*; DHA, dehydroascorbate; *DHAR*, dehydroascorbate reductase; Fd, ferredoxin; *FeSOD*, iron SOD; *GR*, glutathione reductase; GSH, reduced glutathione; GSSG glutathione disulfide; H_2_O_2_, hydrogen peroxide; MDHA, monodehydroascorbate; *MDHAR*, monodehydroascorbate reductase; *MnSOD*, manganese SOD; ^1^O_2_, singlet oxygen; O_2_**^·^**^−^, superoxide radical; O_2_, molecular oxygen; OH**^·^**^−^, hydroxyl radicals; PEG, polyethylene glycol; PGRs, plant growth regulators; *POD*, peroxidase; qRT-PCR, real-time quantitative polymerase chain reaction; ROS, reactive oxygen species; *SOD*, superoxide dismutase; Spd, spermidine.
