# Correction: Zhang, Y., *et al.* Clones of *FeSOD*, *MDHAR*, *DHAR* Genes from White Clover and Gene Expression Analysis of ROS-Scavenging Enzymes during Abiotic Stress and Hormone Treatments. *Molecules* 2015, *20*, 20939–20954

**DOI:** 10.3390/molecules201219850

**Published:** 2015-12-11

**Authors:** Yan Zhang, Zhou Li, Yan Peng, Xiaojuan Wang, Dandan Peng, Yaping Li, Xiaoshuang He, Xinquan Zhang, Xiao Ma, Linkai Huang, Yanhong Yan

**Affiliations:** College of Animal Science and Technology, Sichuan Agricultural University, Chengdu 611130, China; zhangyan1111zy@126.com (Y.Z.); lizhou1986814@163.com (Z.L.); wangxiaoj12@126.com (X.W.); Diana-Peng@hotmail.com (D.P.); 18349237779@163.com (Y.L.); 18728153879@163.com (X.H.); maroar@126.com (X.M.); huanglinkai@sicau.edu.cn (L.H.); yanyanhong3588284@126.com (Y.Y.)

The authors wish to make the following correction to this paper [1]. Due to mislabeling, the following figures:

**Figure 4:**
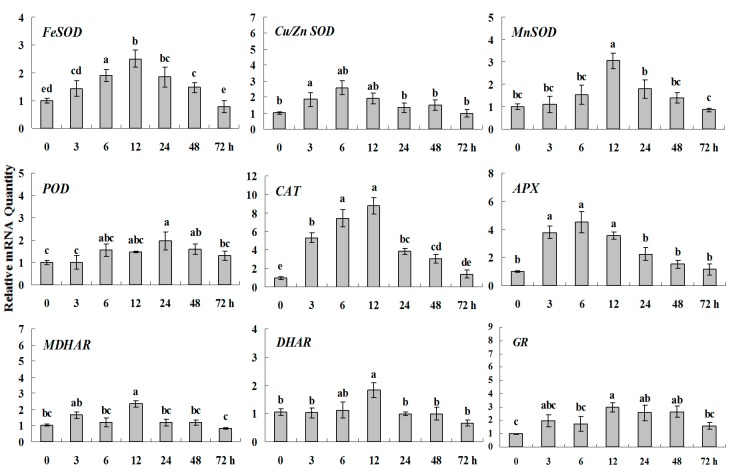
Quantitative real-time RT-PCR was used to analyze of ROS-scavenging enzyme genes expression during drought stress and normalized to β-*actin*. White clover leaves were sampled after 0, 3, 6, 12, 24, 48 and 72 h treatment. Data represent means of three replicates. Error bars representing standard errors and the different letters above the bars represent significant difference (*p* < 0.05).

**Figure 6:**
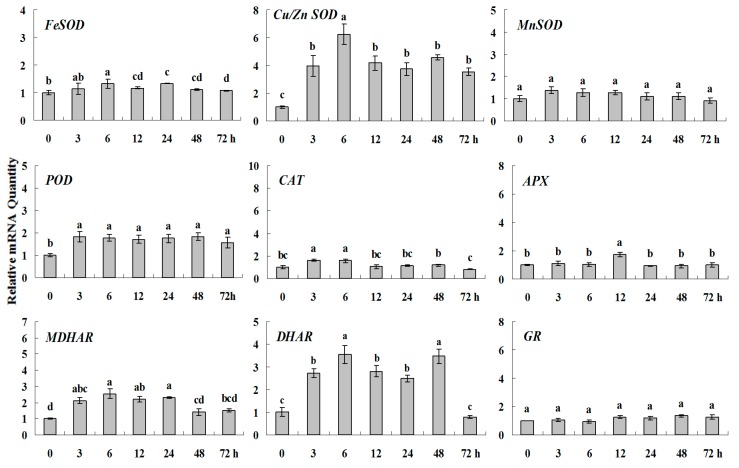
Quantitative real-time RT-PCR was used to analysis of ROS-scavenging enzyme genes expression during heavy metal stress and normalized to β-*actin*. White clover leaves were sampled after 0, 3, 6, 12, 24, 48 and 72 h treatment. Data represent means of three replicates. Error bars representing standard errors and the different letters above the bars represent significant difference (*p* < 0.05).

**Figure 7:**
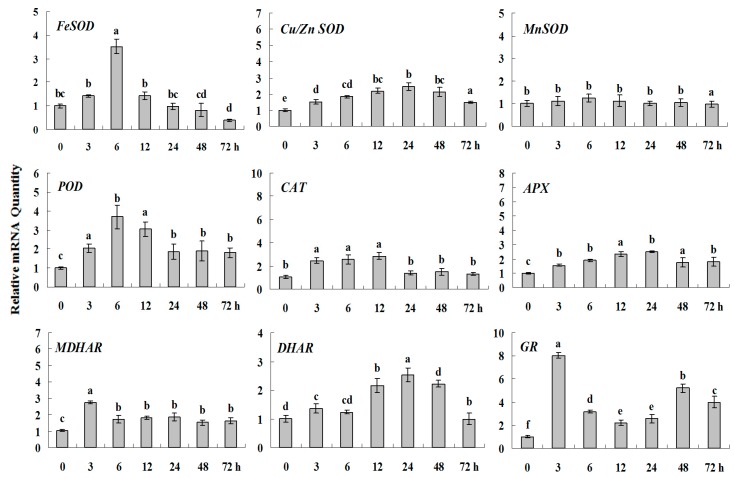
Quantitative real-time RT-PCR was used to analyze of ROS-scavenging enzyme genes expression treatment with ABA and normalized to β-*actin*. White clover leaves were sampled after 0, 3, 6, 12, 24, 48 and 72 h treatment. Data represent means of three replicates. Error bars representing standard errors and the different letters above the bars represent significant difference (*p* < 0.05).

**Figure 8:**
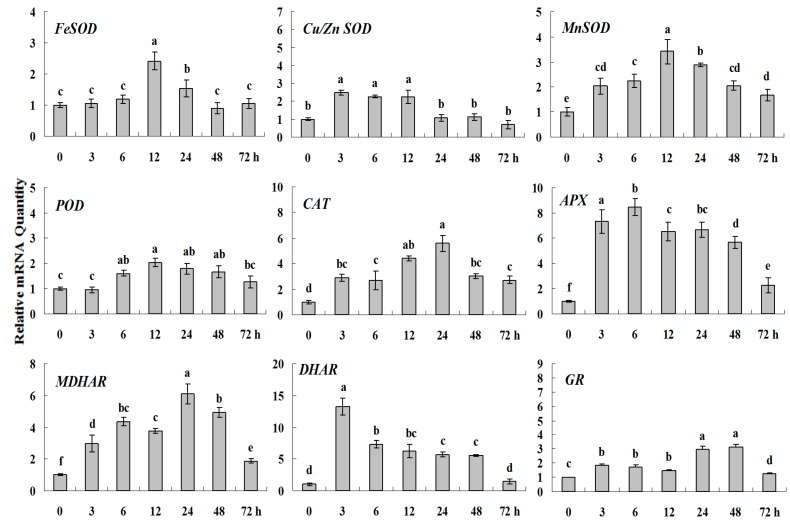
Quantitative real-time RT-PCR was used to analyze of ROS-scavenging enzyme genes expression treatment with Spd and normalized to β*-actin*. White clover leaves were sampled after 0, 3, 6, 12, 24, 48 and 72 h treatment. Data represent means of three replicates. Error bars representing standard errors and the different letters above the bars represent significant difference (*p* < 0.05).

should be replaced by:

**Figure 4:**
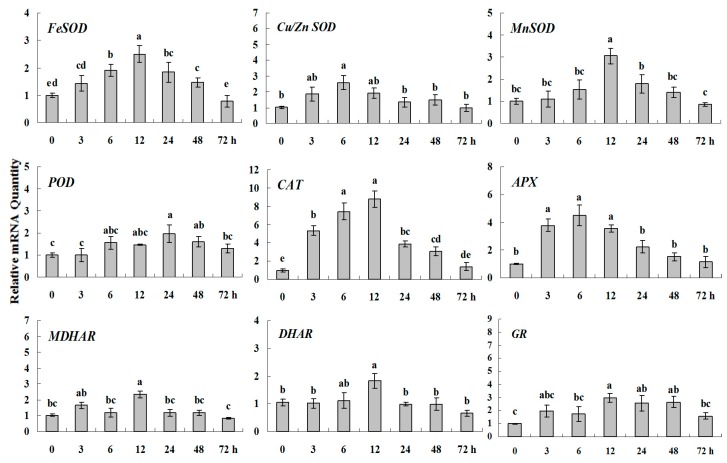
Quantitative real-time RT-PCR was used to analyze of ROS-scavenging enzyme genes expression during drought stress and normalized to β*-actin*. White clover leaves were sampled after 0, 3, 6, 12, 24, 48 and 72 h treatment. Data represent means of three replicates. Error bars representing standard errors and the different letters above the bars represent significant difference (*p* < 0.05).

**Figure 6:**
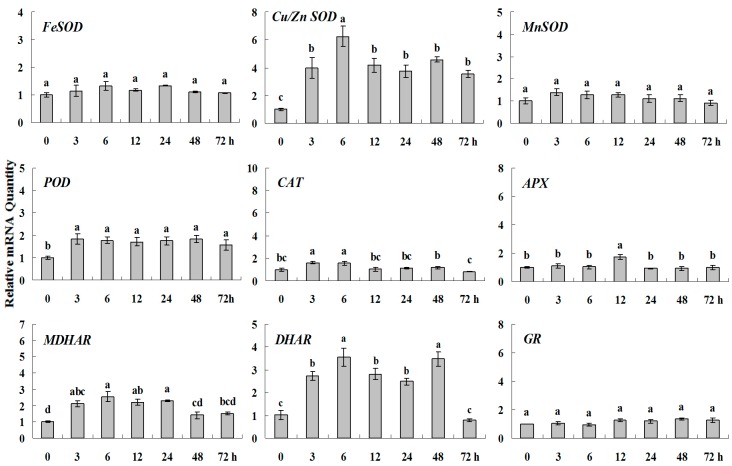
Quantitative real-time RT-PCR was used to analysis of ROS-scavenging enzyme genes expression during heavy metal stress and normalized to β*-actin*. White clover leaves were sampled after 0, 3, 6, 12, 24, 48 and 72 h treatment. Data represent means of three replicates. Error bars representing standard errors and the different letters above the bars represent significant difference (*p* < 0.05).

**Figure 7:**
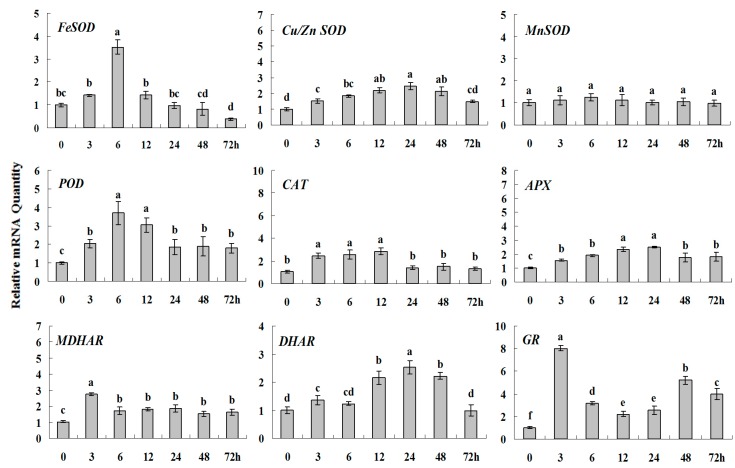
Quantitative real-time RT-PCR was used to analyze of ROS-scavenging enzyme genes expression treatment with ABA and normalized to β*-actin*. White clover leaves were sampled after 0, 3, 6, 12, 24, 48 and 72 h treatment. Data represent means of three replicates. Error bars representing standard errors and the different letters above the bars represent significant difference (*p* < 0.05).

**Figure 8:**
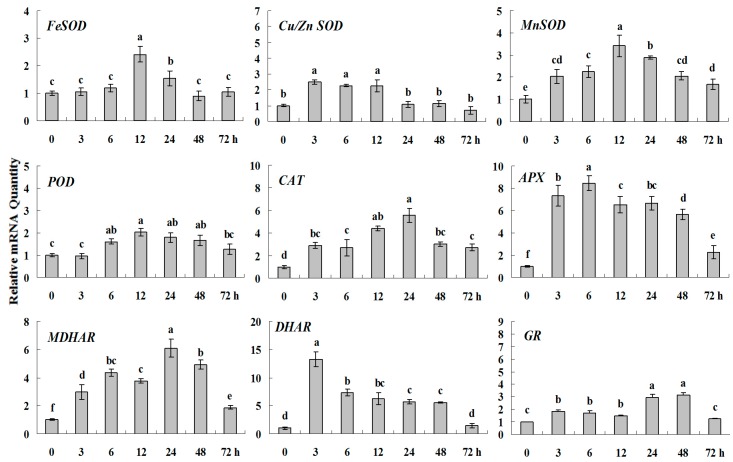
Quantitative real-time RT-PCR was used to analyze of ROS-scavenging enzyme genes expression treatment with Spd and normalized to β*-actin*. White clover leaves were sampled after 0, 3, 6, 12, 24, 48 and 72 h treatment. Data represent means of three replicates. Error bars representing standard errors and the different letters above the bars represent significant difference (*p* < 0.05).

Some of the letters which represented significance were not correct, and accordingly the changes in the figures are as follows: In Figure 4 *FeSOD*, the letters at 6 and 12 h should be changed to b and a, in *Cu/Zn SOD*, the letters at 3 and 6 h should be changed to ab and a. In Figure 6 *FeSOD*, the letters of all treatments except for 6 h should be changed to a. In Figure 7 *Cu/Zn SOD*, the letters of all treatments should be changed to d, c, bc, ab, a, ab and cd, respectively, in *MnSOD*, the letters of all treatments except for 72 h should be changed to a, in *POD*, the letters at 3 and 6 h should be changed to b and a, in *APX*, the letters at 24 and 48 h should be changed to a and b, in *DHAR*, the letters at 48 and 72 h should be changed to b and d. In Figure 8 *APX*, the letters at 3 and 6 h should be changed to b and a, in *GR*, the letters at 72 h should be changed to c. The authors would like to apologize for any inconvenience caused to the readers by these changes.
